# Glioma Tumors’ Classification Using Deep-Neural-Network-Based Features with SVM Classifier

**DOI:** 10.3390/diagnostics12041018

**Published:** 2022-04-18

**Authors:** Ghazanfar Latif, Ghassen Ben Brahim, D. N. F. Awang Iskandar, Abul Bashar, Jaafar Alghazo

**Affiliations:** 1 Faculty of Computer Science and Information Technology, Université du Québec à Chicoutimi, 555 Boulevard de l’Université, Chicoutimi, QC G7H2B1, Canada; ghazanfar.latif1@uqac.ca or; 2 Department of Computer Science, Prince Mohammad bin Fahd University, Khobar 31952, Saudi Arabia; 3 Faculty of Computer Science and Information Technology, Universiti Malaysia Sarawak, Kota Samarahan 94300, Malaysia; dnfaiz@unimas.my; 4 Department of Computer Engineering, Prince Mohammad bin Fahd University, Khobar 31952, Saudi Arabia; abashar@pmu.edu.sa; 5 Department of Electrical and Computer Engineering, Virginia Military Institute, Lexington, VA 24450, USA; alghazojm@vmi.edu

**Keywords:** multi-class Glioma tumors, tumor classification, convolutional neural networks, CNN features

## Abstract

The complexity of brain tissue requires skillful technicians and expert medical doctors to manually analyze and diagnose Glioma brain tumors using multiple Magnetic Resonance (MR) images with multiple modalities. Unfortunately, manual diagnosis suffers from its lengthy process, as well as elevated cost. With this type of cancerous disease, early detection will increase the chances of suitable medical procedures leading to either a full recovery or the prolongation of the patient’s life. This has increased the efforts to automate the detection and diagnosis process without human intervention, allowing the detection of multiple types of tumors from MR images. This research paper proposes a multi-class Glioma tumor classification technique using the proposed deep-learning-based features with the Support Vector Machine (SVM) classifier. A deep convolution neural network is used to extract features of the MR images, which are then fed to an SVM classifier. With the proposed technique, a 96.19% accuracy was achieved for the HGG Glioma type while considering the FLAIR modality and a 95.46% for the LGG Glioma tumor type while considering the T2 modality for the classification of four Glioma classes (Edema, Necrosis, Enhancing, and Non-enhancing). The accuracies achieved using the proposed method were higher than those reported by similar methods in the extant literature using the same BraTS dataset. In addition, the accuracy results obtained in this work are better than those achieved by the GoogleNet and LeNet pre-trained models on the same dataset.

## 1. Introduction

Billions of neurons exist in the human brain, whose main purpose is to process information and control the operations of body organs. The complexity of the human brain is beyond the current body of knowledge. The human brain is made up of three parts, including the cerebrum, brain stem, and cerebellum [1]. The skull is the protective shield around these parts to protect the brain from damage and injuries from external dangers. However, the skull can do little to protect the brain from internal neurological factors. One of the most dangerous internal factors is tumors, which damage the cellular level of the brain, leading to patients’ death. Research has shown that detecting tumors at the early stages along with early intervention tremendously contribute to patients’ survival. This makes images of the brain very important in the diagnosis process of different injuries and tumors. Several imaging technologies exist, such as Computed Tomography (CT), X-ray images, and Magnetic Resonance (MR) images. The rapid advancement of computer technology has a direct relationship with the advancement in imaging technology. MR Imaging (MRI) is the most advanced imaging technology, as it is used to visualize the internal structure and body functionality [2]. With the aid of a powerful magnet, the MRI machine can generate meticulous anatomical information of soft tissues in the different parts of the human body. Already, MRI has proven to be a success in detecting brain tumors and heart abnormalities.

Magnetic fields, radio waves, and other technologies are used to produce images of the brain tissues using MRI technology. Four different types of images (known as modalities) are generated based on the variables of the signal frequency and magnetic field strength. These include Fluid-attenuated inversion recovery (Flair), longitudinal relation time-weighted (T1-weighted), T1-contrasted, and transverse relaxation time-weighted (T2-weighted) [3]. In these images, each of the four types of modalities can be distinguished through color, contrast, and various other variables. For example, T1 is the darker portions of the image; T2 is the brighter portions; Flair shows water and macro-molecules. Due to the differences in the physical properties of the various states of the human body tissues (e.g., bleeding, swelling, inflammation, and tumors), the MRI image can be appropriately used to minutely distinguish among these different states.

Even though medical doctors and clinical technicians have extensive skills and expertise to identify the presence or absence of Glioma tumor in a brain MRI, it takes significant time and effort to actually arrive at a conclusive diagnosis. Apart from time and effort, there may be an increase in the risk of misdiagnosis, if the doctor has many MRI images (causing fatigue) to observe in a short span of time. In order to address these concerns, researchers have resorted to automated diagnostic systems based on image identification and classification. Deep Learning (DL) is one such approach where images can be classified with high accuracy in an automated manner [4]. Moreover, these approaches can also extract features from the images (without manual intervention), which are further utilized to classify MRI images into two categories, namely malignant or benign. Convolutional Neural Networks (CNN) are one the most popular DL techniques that have found immense applications in the field of medical diagnosis [5]. Apart from binary classifications (malignant or benign), the MRI images can also be classified into multiple classes to identify the different types of Glioma tumors. Hence, this research paper proposes an innovative method for the detection of multiple classes (Edema, Necrosis, Enhancing, and Non-enhancing) of Glioma brain tumors from MR images using deep-learning-based features with an SVM classifier. The proposed method achieved higher classification accuracy compared to those reported in previous literature using the same dataset (BraTS).

The organization of the rest of the paper is as follows. Section 2 presents a detailed literature review about the machine-learning-based approaches used in tumor identification. Section 3 describes the proposed tumor classification methodology. Section 4 details the performance results of the proposed model along with a comparative study with existing works. Discussions related to the relevance of the results are presented in Section 5. Finally, in Section 6, conclusions are drawn and future research directions are suggested.

## 2. Literature Review

The task of tumor identification is very complicated and requires specialized knowledge, skills, and analysis techniques to correctly locate the tumor. This task requires capturing a high-resolution image of the internal structure of the brain. Three image modalities are used by doctors to diagnose brain tumors, which are Positron Emission Tomography (PET), Computed Tomography (CT), and Magnetic Resonance Imaging (MRI). MRI uses radio waves, powerful magnets, and computing equipment to capture the details of the brain’s internal structures. Compared to the other modalities, MRI provides better contrast, brightness, and picture details because of the properties of tissue relaxation (T1 and T2), which makes them the preferred method for diagnosis by doctors [6]. However, the diagnosis by doctors and technicians is a slow and lengthy process [7]. Based on different variables such as brightness, contrast, repetition time, and time to echo, the MRI machine produces four different images (T1, T2, T1c, and Flair) [8]. The four scans are utilized in image processing methods for the automatic diagnosis and classification of tumors through feature extraction.

The accuracy of the classification techniques is dependent on the criteria used for feature selection. Various feature extraction techniques have been proposed in recent research, such as Gabor features [9], wavelet transform features, discrete cosine features, discrete Fourier transform features, and statistical features [10,11]. A feature-based automated system for brain MR image classification is proposed in which the tumorous slice classification is presented within the pre-processing stage [12]. Block-based feature extraction is proposed along withRandom Forests (RFs) for the binary classification of MR images to tumorous and normal. Each image is divided into 8 × 8 overlapping blocks in which three Haralick features are extracted; energy, Directional Moment (DM), and Inverse Difference Moment (IDM) from each block. The BraTS 2015 dataset was used, which consists of 274 multi sequence MR images for Glioma patients. Metrics used for validating the results include specificity, sensitivity, Missed Alarm (MA), accuracy, and False Alarm (FA). The results obtained were 94% sensitivity and specificity, 95% accuracy with an error rate of 1%, and 3% spuriously classified as tumorous. However, the study contained several limitations. Firstly, the method only performs the binary classification of the images to tumorous and normal images. Secondly, the method utilizes only three Haralick features due to the division of the image into 8 × 8 overlapping blocks; hence, the processing time is increased. A detailed comparison of discrete cosine features, wavelet transform features, discrete Fourier transform-feature-based classification of MR images into tumorous and non-tumorous, which shows promising results, was performed [13]. However, these experiments were performed using a small dataset consisting of 255 slices.

Another feature-based study was conducted to compare various methods for multi-class Glioma brain tumor classification [14]. The authors used the BraTS dataset and divided the data into 30 volumes for training and 57 for testing. Features were retrieved for the four modalities; T1, T1 contrast-enhanced, T2, and Flair. The features included gradient magnitude, intensity, Laplacian, standard deviation, range, skewness, entropy, kurtosis, and the minimum and maximum in five voxel neighborhoods. All these are commonly used features in brain tumor segmentation and classification. The authors tested with various classifiers available in WEKA. These included Random Forest (RF), Decision Stump, Extra Trees, J.48 Trees, Hoeffding Tree, Conjunctive Rule, Decision Table, Decision Table/Naive Bayes (DTNB), Fuzzy Unordered Rule Induction Algorithm (FURIA), One Rule (OneR), Lazy Family, KStar, Instance-Based Learning (IBL), Locally Weighted Learning (LWL), Functions Family, Linear Discrimination Analysis (LDA), Support Vector Machines (SVMs), Large Linear Classification (LINEAR), Multi-Layer Perceptron (MLP), Fuzzy Lattice Reasoning (FLR), Hyperpipes, and the Input Mapped Classifier. The results showed that RF outperformed all other classifiers for the selected features and achieved an average accuracy of 80.86%. The limitation of this study is evident in which the authors used only statistical features that are general in nature and have been suggested in other brain tumor classification studies. Furthermore, the authors tested for various classifiers within the WEKA software without much novelty in the selection process of the features that would enhance the classification process, as well as the best-performing classifier (RF).

In a different study, the authors proposed a method using features from multi-modal MRI, including structural MRI and Anisotropic and Isotropic components derived from Diffusion Tensor Imaging (DTI) [15]. The proposed method is based on a 3D-supervoxel-based learning method for the segmentation of brain tumors into multi-modal brain images. A variety of features were extracted, including the histogram calculated using a set of Gabor filters with various sizes and orientations, as well as the first-order intensity statistical feature. In their study, the authors used the RF classifier. Two different datasets were considered: the BraTS 2013 dataset was used along with an in-house clinical dataset consisting of 11 multi-modal images of patients [16]. The in-house dataset produced an average detection sensitivity of 86% compared to the 96% achieved in the case of BraTS, while the segmentation results against the ground truth were 0.84 for the in-house dataset and 0.89 for BraTS. This study showed that supervoxels, in general, have a limitation of segmenting small volumes with additional limitations at the tissue boundary, causing an overlap with other tissue types.

The handcrafted feature extraction techniques for brain tumor classification are more prone to over-fitting and may lead to high misclassification rates due to the dependency on the experience of the designer [7,17,18]. Performance improvement of brain tumor classification is still possible through the optimization of deep CNN as a feature extractor and deep CNN as a classifier [4,19]. It is also important to mention the observation that most of the research performed on brain tumor classification is conducted using relatively small datasets.

## 3. Methodology

**Figure 1:**
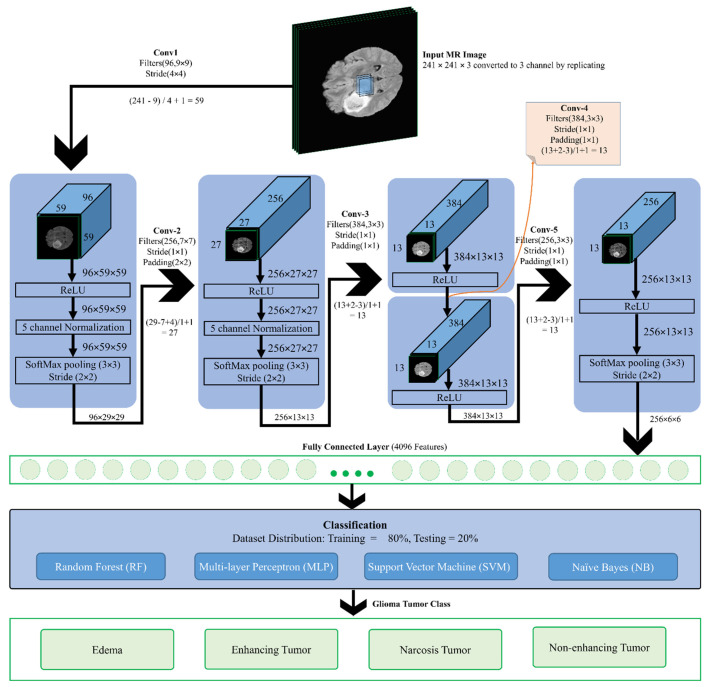
The architecture of the proposed CNN Features based Multiclass Tumor Classification.

The proposed methodology consists of multiple steps. Figure 1 details the proposed architecture for the classification of Glioma tumors into its four classes: (1) Edema, (2) Necrosis, (3) Enhancing, and (4) Non-enhancing. As shown, the MR images of size 241 × 241 (obtained by converting the original image size of 240 × 240) are provided as the input to the proposed CNN model by converting them into three channels. The details of the proposed 17-layer CNN model for feature extraction are presented next. As a result of the proposed CNN architecture, a total of 4096 features are extracted. These features are then used as input to the following four classifiers: Random Forests (RFs), Multi-Layer Perceptron (MLP), Support Vector Machines (SVMs), and Naive Byes (NB), with the goal of classifying the tumor into one of the aforementioned tumor classes.

### 3.1. Proposed Convolutional Network for Feature Extraction

Convolutional Neural Networks (CNNs) are used in deep learning for feature extraction, which automatically entails outperforming other feature extraction techniques. This is done by using filters that glide over the input image to produce what is referred to as a feature map. The produced features are affected by the type of filter being used in the process. The number of image features are affected by the number of filters used. To control the size of the feature map, the parameters such as stride (pixel numbers covered by the filter), zero padding (the process of bordering the image with zeros), and depth (number of filters) need to be controlled.

Since real data are mostly non-linear, thus the ReLU function, which is a non-linear operation, was used. The main task of ReLU is to replace the negative pixel values with zeros, which results in the so-called rectified feature map. To reduce the sensitivity to the network and speed up the training process, a normalization layer is required. This entails the subtraction of the mini-batch mean followed by the division by the standard deviation of the mini-batch. The mini-batch input $B=\left\{x_{1,\ldots \ldots m}\right\}$ with the parameters β and γ, produces the output layer $\left\{y_{i}={BN}_{\gamma ,\beta \;}\left(x_{i}\right)\right\}$, as shown in Equations (1)–(4).

(1) $$\mu_{B}\leftarrow \frac{1}{m}\sum_{i=1}^{m}x_{i}$$

(2) $$\sigma_{B}^{2}\leftarrow \frac{1}{m}\sum_{i=1}^{m}{{(x}_{i}-\;\mu_{B})}^{2}$$

(3) $$x_{i}^{\prime }=\frac{x_{i}-\mu_{B}}{\sqrt{\sigma_{B}^{2}+\;\epsilon }}$$

(4) $$y_{i}\leftarrow \;\gamma x_{i}^{\prime }+\beta \;\equiv {BN}_{\gamma ,\beta \;}\left(x_{i}\right)$$

A pooling layer is used to reduce the spatial dimensions and computational complexity, in addition to addressing the over-fitting problem. Though various pooling functions can be utilized, max pooling is the most widely used and was used in the current model with a filter size of 2 × 2. A Fully Connected Layer (FCL) refers to the fact that each neuron in the consecutive layers is connected to the SoftMax, which can be used for the non-linear combinations of the features.

The proposed convolutional network of 17 layers is used in the CNN feature extraction method for MR image feature extraction, as depicted in Figure 1. The proposed CNN model is a modified version of the LeNet architecture, which has a total of 25 layers [5,20]. The choice of LeNet is due to the fact that it has a good performance on grayscale images, as well as its simplicity compared to GoogleNet, as GoogleNet resulted in over-fitting when tested [21]. A high correlation with neighboring pixels exists for tumorous and non-tumorous pixels in MR images. The output of the convolutional layer is normalized using Local Response Normalization (LRN), which takes the mean of the local pixels, and this method is adopted in AlexNet and the proposed model in this paper.

In the proposed model, the input MR image passes through 96 filters of size 9 × 9 with a stride of 4 × 4. The image is minimized to a size of 96 × 59 × 59. The activation function ReLU, which outputs the input directly if positive or zero otherwise, is then applied to the input function. To stabilize the training, a 5-channel normalization is applied. The gradient is computed based on the maximum value in the window using the SoftMax pooling function of size 3 × 3 with a stride of 2 × 2. Then, 256 filters of size 7 × 7 and a stride of 1 × 1 along with a 2 × 2 padding are applied to the output image of size 96 × 29 × 29. This produces an image of size 256 × 27 × 27, which is again treated with the ReLU function followed by 5-channel normalization preceded by SoftMax pooling to produce an image of size 256 × 13 × 13. Further, 384 filters of size 3 × 3 with stride 1 × 1 and padding 1 × 1 are applied to the output phase to produce an image of size 384 × 13 × 13. The ReLU function is applied three times on the image and then followed by a SoftMax pooling of size 3 × 3 and stride 2 × 2 to output an image of size 256 × 6 × 6. Primitive features are captured using the first layers, which are combined in the later layers in order to form high-level features of the image in preparation for the recognition phase. This phase results in the extraction of 4096 features, which are input to RF, SVM, MLP, and NB classifiers.

### 3.2. Classification

Different types of classifiers were used in this research work, which included SVM, Random Forest, MLP, and Naive Bayes.

*Support Vector Machine (SVM)* is a type of classifier algorithm described by a separate hyperplane. Support Vector Machine’s goal is to discover a hyperplane within N-dimensional space, which helps in classifying the points of data clearly. These points of data are known as the support vector, which is nearer to the hyperplane and affects the hyperplane’s orientation and position. It helps in increasing the classifier’s margin. Points of data that fall on every side of the hyperplane can be assigned to various classes, and the hyperplane’s dimension relies on the number of features. For example, if the number of input features is 2, then the hyperplane is a line, and if the number of input features is 3, then the hyperplane is a 2D plane [22].

*Multilayer Perceptron (MLP)* is a supervised machine learning approach that has its basis the Artificial Neural Network (ANN) architecture [23]. The idea of ANN is borrowed from the structure and functioning of the human brain. As we know, the human brain consists of billions of neurons, which communicate electrical signals to and from the rest of the human body for their proper functioning and control. The basic unit of the human brain (neuron) is modeled artificially as a perceptron in the ANN. A collection of such multiple perceptrons in a row-wise and columnwise arrangement (layers) results in an MLP. At the basic level, there is a single vertical layer at the input, another similar layer at the output, and one (or more) hidden layer in between to realize an MLP. The MLP takes the input data via the input layer and builds a non-linear model of the data to provide an estimate of the desired target variable at the output. Since the layers are inter-connected with each other, the more input data are provided to the MLP, the more it tries to learn the model by adjusting the various weights in the model through the Back-Propagation Algorithm. The strength and the number of inter-connections affect two things, namely the accuracy of the model and its ability to generalize. A balance of over-fitting and under-fitting is desired so that the MLP can perform well in both aspects.

*Random Forest (RF)* is the most common ensemble approach used in classification to construct predictive models. In Random Forest, the model generates a complete forest with decision trees that are not correlated. Then, these decision trees are combined to obtain an accurate prediction. While trees keep growing, more randomness are added to the model by Random Forest. It looks for the best characteristic among various random characteristics instead of looking for the most significant characteristic when a node is splitting. Then, only one random subdivision of the characteristic is considered for the splitting of a node. Random Forest is a widely used algorithm because of its simplicity and better results [24].

*Naive Bayes (NB)* is used to achieve more accurate classifications, and a high training speed based on a random process, which is relied on for local classifiers [10]. It does not need an iterative process for its training and needs only a small amount of training in order to predict the parameter. Furthermore, it has high classification accuracy in terms of the performance results. Bayes’ theorem of probability is the basic theorem used for the Naive Bayes classifier. The main conditional probability in Bayes’ theorem is that an event *W* related to a random class *H* possibly can be computed from theprospect of discovering a specific event in every class and the ultimate prospects of the incident in every class. Assuming *d* belongs to *D* and *Z* classes and *D* is a random value, the probability of *s* related to a class *H* is calculated by:

(5) $$P\left(\left.Z_{h}\right|d\right)=P\left(Z_{h}\right)\frac{P\left(\left.Z_{h}\right|d\right)}{P(d)}$$

### 3.3. Dataset Description

The dataset used in most brain tumor segmentation is the MICCAI BraTS 2018 dataset [16,25]. It is the dataset of choice for most research performed on machine learning and Glioma tumors [2,7,11,14,15,26,27] and many others. The MICCAI BraTS 2018 dataset is especially used in testing the different strategies used in the segmentation of brain tumors in multi-modal images. Further, BraTS 2018 is mainly focused on the MRI method used in surgeries. They are used for the segmentation of specific brain tumors called Glioma tumors. Furthermore, the segmentation is usually performed in the manifestation, shape, and histology. They also ensure that the patient’s life is protected against any mistakes during the medical procedures. As we know, Glioma tumors are considered to be the most widespread brain malignancy in the world. It has been observed that, once the patients is diagnosed with an advanced stage Glioma tumor, he/she is usually left with another two years of life. Therefore, it is imperative that the earlier this tumor is diagnosed, there is a higher chance of extending the survival duration of the patients. In the dataset, for each event, the multi-channel data are made of 4 different 3D MRIs. There are a total of 56 cases; 39 HGG (High-Grade Glioma) and 26 LGG (Low-Grade Glioma). The total number of images for the 56 cases are 40,300 divided into 24,180 images of HGG and 16,120 images of LGG. The images are of size 240 × 240 (which we changed to 241 × 241 for the purpose of our proposed methodology) The dataset contains a total of 4430 tumorous MR images in each sequence type, out of which 1551 are HGG and 2879 are LGG. In addition, the dataset contains a total of 4250 non-tumorous MR images in each sequence type, of which 2169 are HGG and 2081 are LGG. The 4 modalities are Flair, T1-weighted, T1-contrasted, and T2-weighted. The dataset comes with some pre-processing steps already applied such as the re-calculation of the equal ${1mm}^{3}$ resolution, skull-stripping, and a co-registration of all scan cases to magnify the unhealthy tissues. The segmentation of the dataset was performed by experts to obtain the ground truth of the segmentation mask. The dataset also includes patient age and resection status, as well as the overall survival data specified in days.

## 4. Experimental Results

The proposed method is a feature extraction method using the CNN followed by classification using the different classifiers, namely RF, MLP, NB, and SVM. The accuracy results for these four classifiers for multi-class classification are presented in Table 1. As stated in Section 3, the CNN model extracts a total of 4,096 features. As shown in Table 1, for HGG, the average accuracy using the proposed CNN features and RF classifiers ranged from 91.39% to 92.51%. The Naive Bayes classifier with CNN features performed the worst among the four classifiers in this study. The SVM with CNN features performed the best among the four classifiers tested on HGG tumor, achieving an average accuracy between 95.29% and 96.19% with the highest accuracy achieved using the Flair modality. The highest average accuracy for SVM was 96.19% achieved for Flair MR images with a precision of 0.958, a recall of 0.851, and a F1-measure of 0.870.

When applied to LGG MR images, the range of the average accuracy for all four classes was from 74.13% to a maximum of 95.46%. Applying the Naive Bayes classifier with CNN features achieved the highest average accuracy of 77.44% for Flair images, and for the SVM with CNN features, the highest average accuracy was 95.46% using the T2 modality, as shown in Table 1. The highest average accuracy of 95.46% for all four classes was achieved by the SVM with a precision of 0.890, a recall of 0.861, and an F1-measure of 0.889. In conclusion, the SVM classifier used in conjunction with the CNN-extracted features achieved the highest accuracies among all the different classifiers tested in this work. The proposed method outperformed the results of methods reported in previous literature using the same dataset, as further discussed in this section and the Discussions.

**Figure 2:**
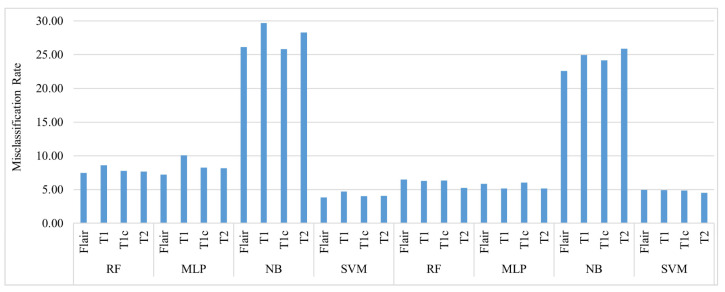
Comparison of the average misclassification of four Glioma Tumor classes using different Classifiers.

Figure 2 shows a comparison of the misclassification of the four Glioma tumor classes using the four classifiers used in this paper. It is evident that for both LGG and HGG, Naive Bayes performed poorly by misclassifying the four classes between 25% and 30% of the time. SVM outperformed the other classifiers for HGG and LGG with a misclassification rate of less than 5% for all classes. For HGG, RF and the MLP had almost the same performance in terms of the misclassification rate; however, RF was slightly better in performance with a misclassification rate of less than 8% for all classes.

## 5. Discussions

The proposed CNN feature-based method was used to classify the four classes of Glioma tumor of the HGG and LGG types. Following the feature extraction step, typical classifiers such as RF, Naive Bayes, SVM, and the MLP were applied, and accuracy-based performance was compared. The CNN features were used for both types of HGG and LGG, and the best accuracy was achieved, as presented in Table 1, with the SVM classifier, where the highest average accuracy of 96.19% was observed for HGG, while using RF, the highest average accuracy was 92.51%; the lowest accuracies were observed with Naive Bayes, where the average accuracy was 74.17%. A similar trend in the results was observed for LGG classification, where the best accuracy was achieved when the SVM classifier was used, as shown in Table 1.

To illustrate the strength of the proposed model in the classification of the four Glioma tumor classes, well-known CNN models such as GoogleNet and LeNet were tested using the BraTS dataset. As can be seen in Table 2, for HGG, the best accuracy was achieved using GoogleNet for the T1c class with an average accuracy of 87.75%, a precision of 0.884, a recall of 0.839, and an F1-measure of 0.860. Compared to an accuracy of 95.98% for the same class using SVM in the proposed model shown in Table 1, the proposed model showed an increase of in the accuracy performance of 8.23% for the same class and even higher for other classes. For LGG, again, GoogleNet outperformed LeNet for the Flair modality with an average accuracy of 85.50%, a precision of 0.867, a recall of 0.722, and an F1-measure of 0.757. Again, comparing that with the result obtained using the proposed model and the SVM classifier, which provided an average accuracy of 95.02% for the same class, showed that the proposed model had an increase of 9.52% and an even higher increase for the other classes.

As shown in Table 3, the proposed technique using CNN features achieved an average accuracy of 95.83% for multi-class classification. When compared with other recent techniques from the literature, it is evident that the proposed technique outperformed the existing approaches as listed in Table 3.

For the purpose of validation, two independent datasets, AANLIB and PIMS, were used [11,28]. The AANLIB dataset is available on the Harvard Medical School website, which consists of 90 Flair modality MR images with 62 non-tumorous and 28 tumorous images [28]. The PIMS MRI dataset consists of 258 T1 modality MR images including 144 normal and 114 tumorous images [11]. The results obtained using these two datasets for the proposed CNN feature-based approach are shown in Table 4. The results were reaffirmed and validated, showing that for both the AANLIB and PIMS datasets, the proposed CNN feature-based approach was in the excellent category by achieving a 100% classification accuracy.

The manual procedure of diagnosis will allow only the diagnosis of patients receiving a brain MRI after all other lab and medical symptoms indicate a possible tumor. This would indicate that early diagnosis is achieved only in rare cases, and in most cases, the diagnosis is performed when the tumor is at later stages and untreatable. If an automatic detection and diagnosis method were developed, in that case, MRI could be a normal procedure in an annual checkup and allow early diagnosis of Glioma brain tumors, which will have these benefits: (1) dramatically decreasing the cost of detection and diagnosis procedures, (2) decreasing the patient’s healthcare costs because early detection will allow for medical procedures and medication at reasonable prices compared to medical procedures performed on patients during a late-stage cancer, (3) reducing the cost of treatment for both hospitals and insurance companies, (4) prolonging the patient’s life and possibly curing the patient of the tumor, and (5) decreasing the healthcare costs at the national level because cancer treatment centers are costly and patient’s waiting time is long. With all the benefits of an automatic detection and diagnosis procedure, computers and servers can work around the clock to go through all the patient’s MR images, flagging any image that shows signs of a Glioma brain tumor. The research community is therefore working on identifying innovative methods for the automatic detection and diagnosis procedures.

## 6. Conclusions

The complexity of diagnosing Glioma brain tumors is apparent from the complexity of the human brain tissue and its anatomy. The manual diagnosis process requires many years of training for both technicians and specialized medical doctors, and even then, it is a time-consuming and costly procedure. Hence, automatic detection and diagnosis methods are proposed and developed for the multiple types of Glioma tumor classification. In this paper, we proposed using a Deep Learning Neural Network to extract the features of the MR image, which are then given as the input to various classifiers (NB, RF, SVM, and MLP), with the SVM classifier achieving the highest accuracy. With the proposed technique, a 96.19% accuracy was achieved for the HGG type with the Flair modality and 95.46% for the LGG tumor type with the T2 modality. Compared to similar methods using the BraTS dataset, the proposed technique produced far better results. Well-known CNN models were used on the same dataset to show the strength of the proposed model. The well-known CNN models such as GoogleNet and LeNet were used with GoogleNet outperforming LeNet for both LGG and HGG. GoogleNet produced an accuracy of 87.75% for T1c HGG and 85.5% for Flair LGG. However, the proposed model outperformed the well-known CNN models and other models in the extant literature, with the SVM classifier producing an average accuracy of 95.83%. Future work will include developing and enhancing the proposed technique detailed in this paper to cover not only the Glioma tumor classification, but other medical conditions such as skin cancers, breast cancers, lung cancers, and others.

## Figures and Tables

**Table 1:** Comparison of Multi-class Glioma Tumor Classification using CNN Features with typical Classifiers.

Glioma Type	Classifier	Modality	Individual Accuracies				Average Measures			
			Necrosis	Edema	Non-Enhancing	Enhancing	Accuracy	Precision	Recall	F1-Measure
HGG	RF	Flair	90.41	99.29	90.31	90.02	92.51	0.930	0.793	0.805
		T1	88.53	99.32	88.66	89.05	91.39	0.914	0.772	0.792
		T1c	90.90	99.22	88.89	89.86	92.22	0.795	0.781	0.787
		T2	90.86	99.32	89.34	89.89	92.35	0.927	0.788	0.808
	MLP	Flair	89.99	99.13	91.06	90.86	92.76	0.928	0.778	0.928
		T1	86.78	99.42	86.65	86.88	89.93	0.899	0.750	0.899
		T1c	90.25	99.22	88.05	89.41	91.73	0.918	0.768	0.918
		T2	89.99	99.29	89.28	88.86	91.85	0.919	0.769	0.919
	NB	Flair	65.08	89.89	69.97	70.49	73.86	0.720	0.782	0.715
		T1	57.79	89.73	66.28	67.57	70.34	0.696	0.724	0.671
		T1c	70.36	88.53	68.22	69.58	74.17	0.715	0.767	0.708
		T2	60.97	89.21	67.64	68.97	71.70	0.711	0.751	0.693
	**SVM**	Flair	94.95	99.45	95.30	95.04	**96.19**	**0.958**	**0.851**	**0.870**
		T1	93.39	99.25	94.10	94.43	95.29	0.915	0.830	0.848
		T1c	95.21	99.42	94.66	94.62	95.98	0.918	0.844	0.861
		T2	94.53	99.38	94.98	94.72	95.90	0.956	0.833	0.849
LGG	RF	Flair	93.83	100	90.74	89.56	93.53	0.812	0.801	0.804
		T1	91.78	100	91.76	91.32	93.72	0.824	0.808	0.810
		T1c	93.83	100	90.00	90.88	93.68	0.816	0.791	0.800
		T2	93.69	100	92.65	92.65	94.75	0.825	0.811	0.815
	MLP	Flair	92.21	99.56	93.24	91.62	94.15	0.942	0.792	0.942
		T1	92.50	99.85	94.85	92.21	94.85	0.949	0.799	0.949
		T1c	92.65	99.85	92.65	90.74	93.97	0.940	0.790	0.939
		T2	93.97	99.85	92.94	92.50	94.82	0.845	0.798	0.948
	NB	Flair	72.10	99.71	73.24	64.71	77.44	0.735	0.727	0.726
		T1	65.49	100	75.88	58.82	75.05	0.748	0.679	0.705
		T1c	66.81	100	75.44	61.18	75.86	0.740	0.687	0.707
		T2	67.11	100	73.68	55.74	74.13	0.737	0.682	0.702
	**SVM**	Flair	93.82	99.93	92.57	93.75	95.02	0.870	0.860	0.864
		T1	93.82	99.93	92.57	94.04	95.09	0.877	0.861	0.868
		T1c	93.82	99.93	92.50	94.34	95.15	0.873	0.854	0.862
		T2	94.24	99.93	92.72	94.93	**95.46**	**0.890**	**0.861**	**0.889**

**Table 2:** Multiclass Glioma Tumor using other well-known CNN models (GoogleNet and LeNet) for brain MR images.

Glioma Type	CNN Model	Modality	Individual Accuracies				Average Measures			
			Necrosis	Edema	Non-Enhancing	Enhancing	Accuracy	Precision	Recall	F1-Measure
HGG	LeNet	Flair	85.66	97.99	73.55	74.35	82.89	0.811	0.766	0.787
		T1	70.43	97.99	67.87	69.94	76.56	0.734	0.817	0.770
		T1c	75.50	98.32	67.39	71.91	78.28	0.772	0.773	0.768
		T2	76.07	97.99	73.41	77.87	81.33	0.789	0.825	0.805
	GoogleNet	Flair	74.73	97.99	75.93	77.64	81.57	0.791	0.828	0.809
		T1	76.02	96.64	76.13	76.09	81.22	0.801	0.801	0.801
		T1c	86.77	97.32	80.88	86.03	**87.75**	**0.884**	**0.839**	**0.860**
		T2	80.51	99.33	74.82	79.18	83.46	0.819	0.826	0.822
LGG	LeNet	Flair	75.63	98.25	82.40	74.33	82.65	0.765	0.714	0.726
		T1	70.29	98.25	70.71	66.49	76.43	0.776	0.707	0.733
		T1c	66.79	98.25	64.57	61.11	72.68	0.702	0.767	0.729
		T2	76.32	98.25	64.57	74.43	78.39	0.793	0.717	0.751
	GoogleNet	Flair	81.54	98.25	82.55	79.67	**85.50**	**0.867**	**0.722**	**0.757**
		T1	80.60	100.00	78.02	72.33	82.74	0.813	0.810	0.811
		T1c	79.24	100.00	71.88	71.86	80.75	0.765	0.868	0.811
		T2	76.12	98.25	75.39	71.63	80.35	0.763	0.859	0.807

**Table 3:** Comparison of the proposed method for Glioma Tumor Classification with the latest literature techniques.

Method	Dataset Name	Accuracy
Proposed Method (CNN features from Model 1, SVM as the classifier)	BraTS	**95.83%**
Texture Features from Supervoxels and Random Forest as the Classifier, 2018 [15]	BraTS	80%
Ten Statistical Features and Random Forest as the Classifier, 2019 [14]	BraTS	80.85%
Dual-Path Residual Convolutional Neural Network, 2020 [26]	BraTS	84.90%
Deep CNN with Extensive Data Augmentation, 2019 [27]	BraTS	94.58%

**Table 4:** Experimental Results for Validation Datasets (PIMS-MRI and AANLIB) using the proposed CNN feature-based method New validation results table.

Dataset	Classifier	Accuracy	Precision	Recall	F-Measure
AANLIB (two-class dataset)	RF	100	1	1	1
	MLP	94.12	0.923	1	0.96
	SVM	100	1	1	1
	NB	88.24	0.857	1	0.923
PIMS-MRI (two-class dataset)	RF	100	1	1	1
	MLP	94.23	0.906	1	0.951
	SVM	100	1	1	1
	NB	76.47	0.766	0.765	0.761

## Data Availability

The dataset used in this research work was taken from the public domain (University of Pennsylvania) at: https://www.med.upenn.edu/sbia/brats2018/data.html [Last accessed: 12 March 2022].
